# Human Amniotic Epithelial Stem Cells Promote Colonic Recovery in Experimental Colitis via Exosomal MiR‐23a–TNFR1–NF‐κB Signaling

**DOI:** 10.1002/advs.202401429

**Published:** 2024-10-08

**Authors:** Yaohui Kou, Jinying Li, Yingyi Zhu, Jia Liu, Ruizhe Ren, Yuanqing Jiang, Yunyun Wang, Chen Qiu, Jiayi Zhou, Zhuoheng Yang, Tuoying Jiang, Jianan Huang, Xiangyi Ren, Shiguang Li, Cong Qiu, Xiyang Wei, Luyang Yu

**Affiliations:** ^1^ Key Laboratory of Cardiovascular Intervention and Regenerative Medicine of Zhejiang Province of Sir Run Run Shaw Hospital MOE Laboratory of Biosystems Homeostasis & Protection of College of Life Sciences Zhejiang University Hangzhou Zhejiang 310058 China; ^2^ College of Life Sciences‐iCell Biotechnology Regenerative Biomedicine Laboratory Zhejiang University‐Lishui Joint Innovation Center for Life and Health Lishui Zhejiang 323010 China; ^3^ Eye Center the Second Affiliated Hospital School of Medicine Zhejiang Provincial Key Laboratory of Ophthalmology Zhejiang Provincial Clinical Research Center for Eye Diseases Zhejiang Provincial Engineering Institute on Eye Diseases Zhejiang University Hangzhou Zhejiang 310009 China; ^4^ Department of Obstetrics Women's Hospital School of Medicine Zhejiang University Hangzhou Zhejiang 310006 China; ^5^ Department of General Surgery Sir Run Run Shaw Hospital Zhejiang University School of Medicine Liangzhu Laboratory Zhejiang University Hangzhou Zhejiang 310012 China

**Keywords:** colitis, exosome, human amniotic epithelial stem cells, hydrogel, MiRNA

## Abstract

Inflammatory bowel disease (IBD), including ulcerative colitis and Crohn's disease, manifests as chronic intestinal inflammation with debilitating symptoms, posing a significant burden on global healthcare. Moreover, current therapies primarily targeting inflammation can lead to immunosuppression‐related complications. Human amniotic epithelial stem cells (hAESCs), which exhibit low immunogenicity and ethical acceptability, have gained attention as potential therapeutics. In this study, it is demonstrated that their encapsulation in a hydrogel and administration via anal injection enhanced the colonic mucosal barrier repair in a murine colitis model induced by dextran sodium sulfate during the recovery phase. The underlying mechanism involved the release of exosomes from hAESCs enriched with microRNA‐23a‐3p, which post‐transcriptionally reduced tumor necrosis factor receptor 1 expression, suppressing the nuclear factor‐κB pathway in colonic epithelial cells, thus played a key role in inflammation. The novel approach shows potential for IBD treatment by restoring intestinal epithelial homeostasis without the immunosuppressive therapy‐associated risks. Furthermore, the approach provides an alternative strategy to target the key molecular pathways involved in inflammation and promotes intestinal barrier function using hAESCs and their secreted exosomes. Overall, this study provides key insights to effectively treat IBD, addresses the unmet needs of patients, and reduces related healthcare burden.

## Graphical abstract

Encapsulated in a hydrogel and administered via anal injection, human amniotic epithelial stem cells (hAESCs) promote colonic recovery in colitis. This therapeutic effect is mediated by exosomes from hAESCs enriched with microRNA‐23a‐3p, which limit tumor necrosis factor ‐nuclear factor κB signaling and consequently improve dysfunction of intestinal epithelial barrier. This study opens up new avenues for developing effective treatments for inflammatory bowel diseases.

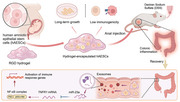

## 1 Introduction

Inflammatory bowel disease (IBD) is characterized by persistent inflammation of the gastrointestinal tract and recurring symptoms, such as abdominal pain, diarrhea, anemia, and weight loss.^[^ ^1^ ^]^ IBD onset is associated with aberrant immune responses, marked by impaired pathogen resistance, recruitment of immune cells to the lamina propria, and excessive release of pro‐inflammatory cytokines. These factors collectively lead to intestinal epithelial injury, compromising the integrity of the intestinal barrier.^[^ ^2^ ^]^ Damage or dysfunction of the intestinal epithelial barrier further exacerbates intestinal inflammation, leading to ulcer formation and various complications, such as fibrosis and fistulas.^[^ ^3^ ^]^

Current therapeutic strategies for IBD mainly focus on reducing inflammation either through non‐specific suppression of the immune system or by targeting specific molecules involved in inflammation.^[^ ^4^, ^5^ ^]^ However, these strategies may increase the susceptibility to other infections and malignancies owing to the potential risks associated with prolonged immunosuppressive treatment. Therefore, new strategies for intestinal epithelial restoration without immunosuppression are needed for IBD treatment.^[^ ^6^ ^]^

Recently, regenerative medicine and cell‐based therapies have emerged as promising options for IBD treatment. Specifically, human amniotic epithelial stem cells (hAESCs) have gained attention in both preclinical and clinical studies. hAESCs are derived from the human amniotic membrane and possess several beneficial properties, such as low immunogenicity, absence of tumorigenicity, easy availability, and high ethical acceptability.^[^ ^7^ ^]^ Various animal studies have shown the therapeutic potential of hAESCs for inflammation‐related injuries, including spinal cord injuries,^[^ ^8^ ^]^ lung injuries,^[^ ^9^, ^10^ ^]^ liver injuries,^[^ ^11^ ^]^ acute kidney failure,^[^ ^12^, ^13^, ^14^ ^]^ wound healing,^[^ ^15^, ^16^ ^]^ pressure ulcers,^[^ ^17^ ^]^ and intrauterine adhesion.^[^ ^18^, ^19^, ^20^ ^]^

**Scheme 1:**
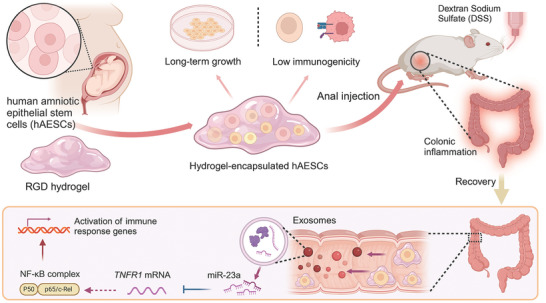
Schematic overview of the overall experimental design to investigate the therapeutic effects of human amniotic epithelial stem cells (hAESCs) encapsulated in arginine–glycine–aspartic acid (RGD) hydrogels in dextran sodium sulfate (DSS)‐induced colitis model mice.

In this study, we aimed to investigate the therapeutic potential of hAESCs for colitis using a dextran sodium sulfate (DSS)‐induced murine colitis model that closely mimicked the key aspects of IBD in humans (**Scheme** **1**). We found that hAESCs showed healing properties by releasing exosomal microRNA (miR)‐23a‐3p, which upon transfer to recipient cells, restored the intestinal epithelial functions under inflammation via the tumor necrosis factor (TNF)/TNF receptor 1 (TNFR1)/nuclear factor (NF)‐κB pathway.

## 2 Results

### 2.1 Optimization of the Hydrogel‐Encapsulated hAESC Composite System

**Figure 1:**
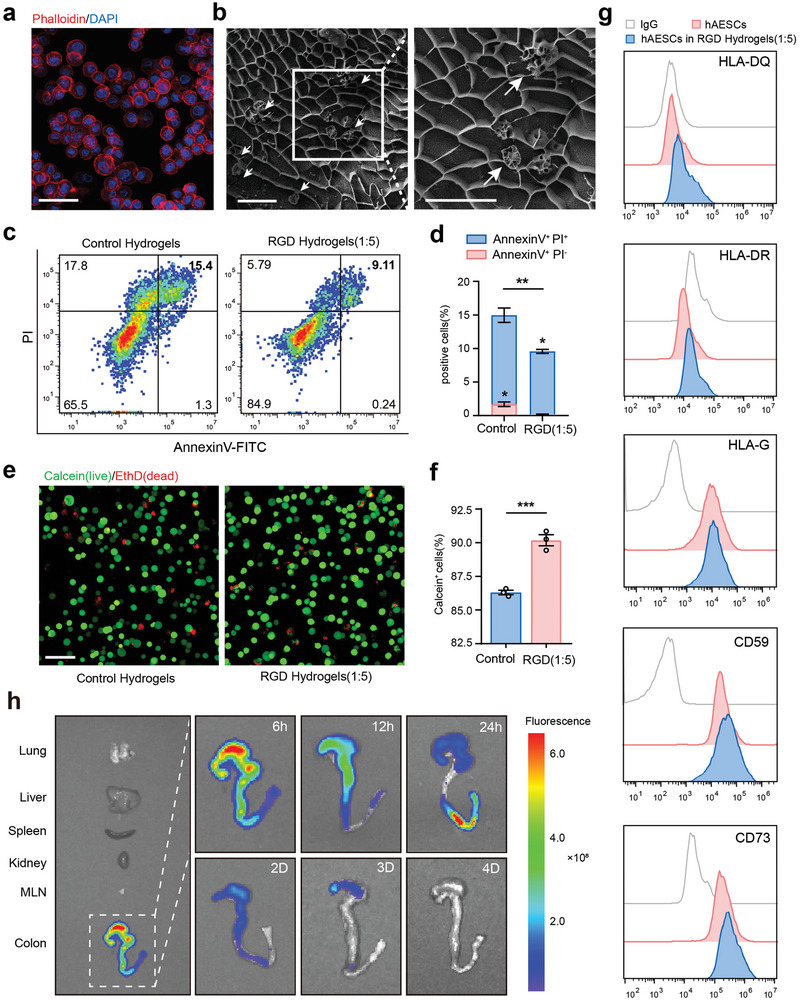
Biocompatibility of high‐concentration arginine–glycine–aspartic acid (RGD) hydrogels. a) Representative fluorescence image of phalloidin staining (red) showing the morphology of human amniotic epithelial stem cells (hAESCs) encapsulated in high‐concentration RGD hydrogels. Scale bars = 100 µm. b) Representative scanning electron microscopy image of the hydrogel‐encapsulated hAESCs composite system. White arrows indicate the hAESCs cultured in hydrogels. Scale bars = 50 µm. c, d) Flow cytometric analysis of hAESCs labeled with annexin V‐fluorescein isothiocyanate (FITC) and propidium iodide (PI) after three days of culture in hydrogels. Cell populations were marked as live (double‐negative), early apoptotic (annexin V‐positive and PI‐negative), apoptotic (annexin V‐positive), and dead (double‐positive) c). Quantification summary is shown in (d). e, f) Calcein (live)/EthD (dead) staining to assess the viability of hAESCs cultured in hydrogels for over three days (e). Quantification summary is shown in (f). Scale bars = 100 µm. g) Flow cytometry analysis of HLA‐DQ, HLA‐DR, HLA‐G (with 10 ng mL^−1^ interferon [IFN]‐γ treatment for 72 h), CD59, and CD73 levels in hAESCs with or without hydrogel culture for 72 h. h) Detection of RGD hydrogel degradation in the intestine. Near‐infrared (NIR) imaging of the fluorescent dye‐labeled hydrogel was performed following anal injections. NIR signals were captured from five main organs (lungs, liver, spleen, kidneys, and colon) and corresponding mesenteric lymph nodes (MLNs) at indicated time points post‐injection. Data are represented as the mean ± standard error of the mean (SEM). Unpaired *t*‐test was used for analysis. n = 3. **p < 0.01 and ***p < 0.001.

The efficiency of therapeutic cell delivery to the desired destination greatly depends on the mode of administration. To optimize the administration method for anal injection considering the epithelial properties of hAESCs (Figure S1a–c, Supporting Information), we designed a hydrogel‐encapsulated hAESC composite system. Phalloidin staining (**Figure** **1**a) and scanning electron microscopy (Figure 1b) revealed that hAESCs maintained their spherical morphology and were scattered randomly throughout the porous structure of the hydrogel matrix.

To identify the optimal hydrogel type, we conducted a cell counting kit‐8 assay. Notably, synthetic arginine–glycine–aspartic acid (RGD) hydrogels promoted higher cell proliferation than the traditional in vitro cell culture methods. This effect was especially prominent at the 5‐times dilution ratio and surpassed that at all other dilution ratios (Figure S1d, Supporting Information). Apoptosis (Figure 1c,d) and live/dead cell staining (Figure 1e,f) assays confirmed that the hAESCs cultured with RGD hydrogels at 5‐times dilution remained viable for three days compared to those cultured with the control hydrogels. Moreover, stability of the cellular characteristics of hAESCs cultured within RGD hydrogels was confirmed via flow cytometry analysis (Figure 1g; Figure S1e, Supporting Information). hAESCs cultured within RGD hydrogels were positive for the typical epithelial markers, such as CD326 (EpCAM) and CD49f, but negative for mesenchymal markers, such as CD90 and CD105. Moreover, RGD hydrogels preserved the immune properties of hAESCs, as indicated by the low HLA‐DQ and HLA‐DR levels but high HLA‐G, CD59, and CD73 levels upon interferon (IFN)‐γ stimulation. CD73 exerts crucial immunosuppressive effects at the maternal–fetal interface in humans.^[^ ^21^ ^]^

By conjugating a fluorescent dye to 5‐times diluted RGD hydrogel, we evaluated the duration of RGD hydrogel retention in the colon. As shown in Figure 1h, the dye‐labeled hydrogel was predominantly detected locally in the large bowel post‐anal injection and gradually diminished over time, persisting in the colon for at least three days.

Collectively, these data suggest that RGD hydrogels at 5‐times dilution are suitable for hAESC culture, without affecting their biological properties. Hydrogel retention in the colon for at least three days further confirmed its suitability for anal injection and delivery of therapeutic cells.

### 2.2 hAESCs Promote Colonic Recovery in DSS‐Induced Colitis Model Mice

**Figure 2:**
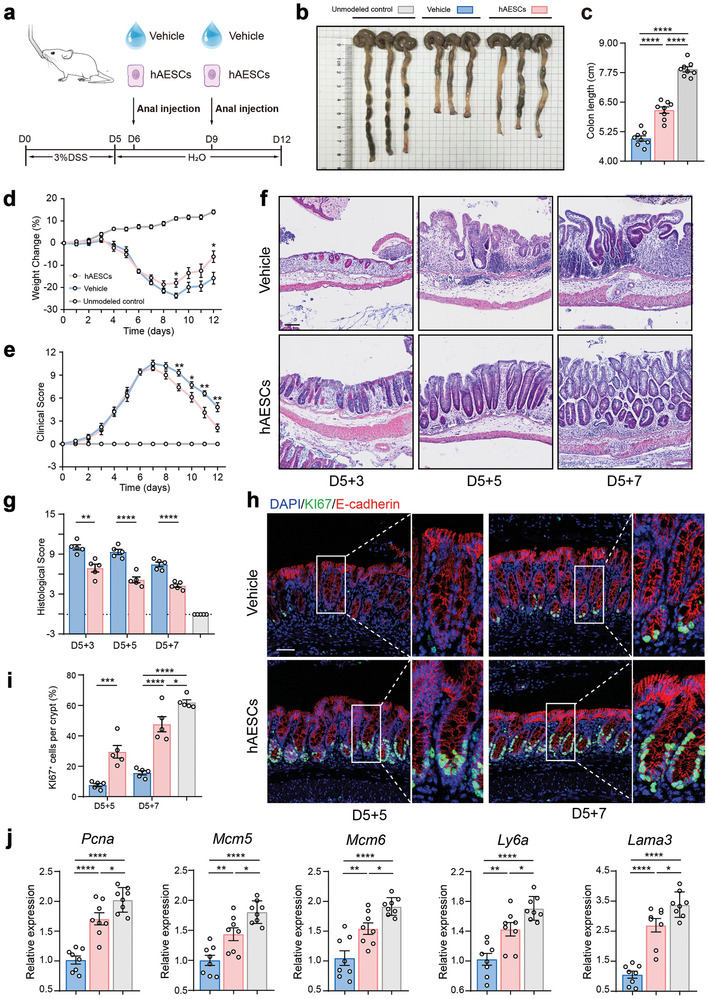
Enhanced recovery of dextran sodium sulfate (DSS)‐induced colitis model mice after hAESC treatment. a) Schematic representation of the treatment protocol for the DSS‐ induced colitis model mice. b, c) Visual assessment of colon morphology (b) and quantification of colon length on day 12 (c). n = 8. d, e) Changes in body weight (d) and disease activity index (DAI) (e) during DSS‐induced colitis induction and recovery in mice. n = 8. f, g) Hematoxylin and eosin (H&E) staining of colon tissue sections (f) and assessment of histological scores (g). Scale bar = 100 µm. n = 5. h–j) Evaluation of regenerated epithelial cells in the colonic mucosa of mice with DSS‐induced colitis with or without hAESC treatment via Ki67 (green) immunofluorescence staining (h). Quantification summary is shown in (i). Quantitative reverse transcription‐polymerase chain reaction (qRT‐PCR) analysis of the indicated genes (j). Scale bars = 50 µm. Data are represented as the mean ± SEM. Unpaired *t*‐test or one‐way analysis of variance (ANOVA) followed by Tukey's post‐hoc test was used for analysis. n = 5–8. ****p < 0.0001, ***p < 0.001, **p < 0.01, and *p < 0.05.

Next, we investigated the roles of hAESCs in colonic inflammation using a well‐established mouse model of colitis. To induce colitis, mice were challenged with DSS in drinking water for five days, followed by seven days of consuming regular drinking water to facilitate recovery. Then, hydrogel‐encapsulated hAESCs were administered via anal injections on days 6 and 9, specifically targeting the areas with peak epithelial damage caused by colitis (**Figure** **2**a).

Intriguingly, addition of hAESCs significantly improved colitis recovery in DSS‐induced mice compared to that in their littermate controls that only received hydrogel injections. This improvement was evident from the reduced colon shortening (Figure 2b,c), rapid recovery of body weight after DSS withdrawal (Figure 2d), and marked decrease in the disease activity index from day 9 (Figure 2e). Additionally, hAESC infusion decreased the histological scores during the recovery phase (Figure 2f,g; Figure S2a, Supporting Information). Notably, no significant sex‐related disparities were observed in the hAESC‐mediated alleviation of colitis in DSS‐induced mice (Figure S2c–g, Supporting Information).

Colonic epithelium undergoes periodic regeneration, typically every 3–5 days.^[^ ^22^ ^]^ During this process, proliferative epithelial cells originate from the base of crypts, migrate upward, differentiate into mature cells, and ultimately replace their aged or damaged counterparts via apoptosis or physiological shedding.^[^ ^23^ ^]^ However, exposure to DSS inhibited colonic epithelial cell proliferation due to the hyperactivation of the immune response and disruption of the colonic structure. This effect was notably alleviated by the anal injection of hAESCs, as indicated by the significant increase in the number of KI67^+^ cells per crypt (Figure 2h,i; Figure S2b, Supporting Information). Meanwhile, the infusion of hAESCs effectively restored cell proliferation and levels of regeneration marker genes, such as *Pcna*, *Mcm5, Mcm6, Ly6a*, and *Lama3*, in colonic epithelial cells. Levels of these genes were initially suppressed by DSS administration (Figure 2j). In summary, these findings suggest that hAESCs protect against DSS‐induced murine colitis by promoting mucosal repair following injury.

### 2.3 hAESCs Alleviate the Abnormal Immune Response and Colonic Epithelium Damage in Mice Following DSS Treatment

**Figure 3:**
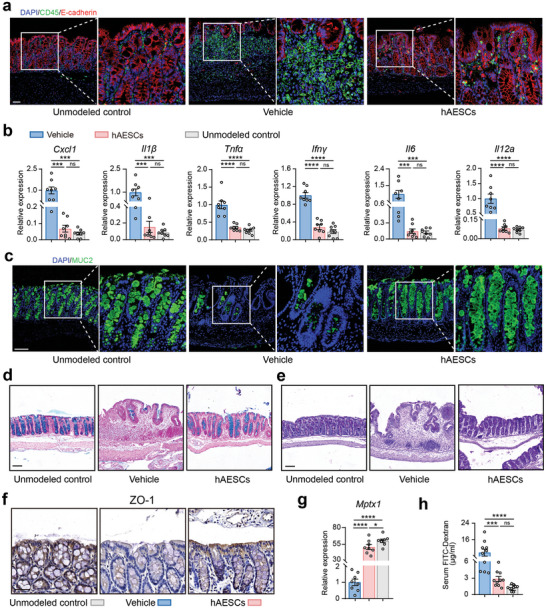
Mitigation of mucosal inflammation and epithelial barrier injury in the colon of DSS‐ induced colitis model mice by hAESC treatment. a,b) Assessment of inflammatory cell infiltration in the colonic mucosa of mice with DSS‐induced colitis with or without hAESC treatment via CD45 (green) immunofluorescence staining (a) and qRT‐PCR analysis of the indicated genes (b). Scale bars = 50 µm. n = 8. c–e) Assessment of mucus barrier in the colon of mice with DSS‐induced colitis with or without hAESC treatment via mucin 2 (MUC2; green) immunofluorescence (c), Alcian blue (d), and periodic acid‐Schiff (e) staining. Scale bars = 100 µm. f) Zonula occludens‐1 (ZO‐1) immunohistochemical staining of the epithelial cells in the colonic mucosa of mice with DSS‐induced colitis. Scale bars = 50 µm. g) Quantification of the mRNA expression levels of the indicated genes in the colonic tissues of mice with DSS‐induced colitis. n = 8. h) Serum FITC‐dextran levels in mice on day 12. n = 8. Data are represented as the mean ± SEM. One‐way ANOVA followed by Tukey's post‐hoc test was used for analysis. ****p < 0.0001, ***p < 0.001, and *p < 0.05; ns, not significant (*p* > 0.05).

Development of colitis is initiated by the abnormal activation of the immune response and uncontrolled production of inflammatory cytokines, leading to the disruption of the intestinal barrier, intestinal dyshomeostasis, and destruction of mucosal integrity.^[^ ^2^ ^]^ Here, CD45^+^ staining revealed greater immune cell infiltration in the colons of the littermate control mice than in the colons of the hAESCs‐treated mice (**Figure** **3**a). To further investigate the effects of hAESCs treatment, we performed quantitative analysis of pro‐inflammatory cytokines and chemokines in colon explants using quantitative reverse transcription‐polymerase chain reaction (qRT‐PCR). mRNA levels of pro‐inflammatory cytokines and chemokines (C‐X‐C motif chemokine ligand [*Cxcl*]‐*1*, interleukin [*Il*]‐*1β*, *Tnfα Ifnγ, Il6*, and *Il12a*) were significantly decreased after administration of hAESCs to mice receiving DSS (Figure 3b). This suggests the therapeutic potential of hAESCs to reduce inflammation and promote recovery of colonic epithelium in colitis.

Mucosal barrier of the intestine plays a crucial role in protecting against microorganisms and maintaining intestinal homeostasis. Impairment of the mucus layer exposes the intestinal epithelium to microorganisms and pathogens, leading to the onset of various gastrointestinal disorders, mainly IBD.^[^ ^24^ ^]^ Levels of mucin 2 (MUC2), a predominant structural protein of the colonic mucus layer, were reduced in mice with DSS‐induced colitis; however, their levels were restored after hAESCs treatment (Figure 3c). mRNA levels of anterior gradient 2 protein, which is involved in MUC biosynthesis,^[^ ^25^ ^]^ were increased after administration of hAESCs following DSS challenge, suggesting the preservation of the mucus barrier. Similar to the levels of MUC2 and other components of the intestinal barrier,^[^ ^24^ ^]^ levels of trefoil factor 3 were increased in hAESCs‐treated mice, indicating intestinal epithelial repair (Figure S3a, Supporting Information). These findings were confirmed by Alcian blue and periodic acid‐Schiff staining, which revealed relatively intact MUCs spread throughout the colon epithelia in hAESCs‐treated mice (Figure 3d,e).

Epithelial tight junctions are essential to maintain the integrity of the intestinal barrier.^[^ ^26^ ^]^ Downregulation of tight junction protein expression compromises the intestinal epithelial barrier integrity and leads to increased intestinal permeability in colitis.^[^ ^27^ ^]^ Therefore, expression levels of the tight junction protein, zonula occludens‐1, were examined by immunohistochemistry (IHC) in this study. Zonula occludens‐1 levels were significantly increased in the colonic epithelial cells of hAESCs‐treated colitis model mice (Figure 2f). Upregulation of other tight junction proteins, including *Mptx1, Occludin*, and *Claudin‐1*, was validated via RT‐qPCR analysis (Figure 2g; Figure S3b, Supporting Information). hAESCs‐treated colitis model mice exhibited reduced plasma levels of fluorescein isothiocyanate (FITC)‐dextran compared to the vehicle control mice after oral gavage of FITC‐dextran (Figure 3h), indicating that hAESCs injection restores the intestinal permeability in DSS‐induced colitis mice.

Collectively, these results highlight the potential of hAESCs to alleviate mucosal inflammation, restore the mucus barrier after injury, and improve the disrupted epithelial tight junctions, thereby ameliorating colitis in mice following DSS administration.

### 2.4 hAESCs Protect Against DSS‐Induced Colitis by Downregulating the NF‐κB Signaling Pathway

**Figure 4:**
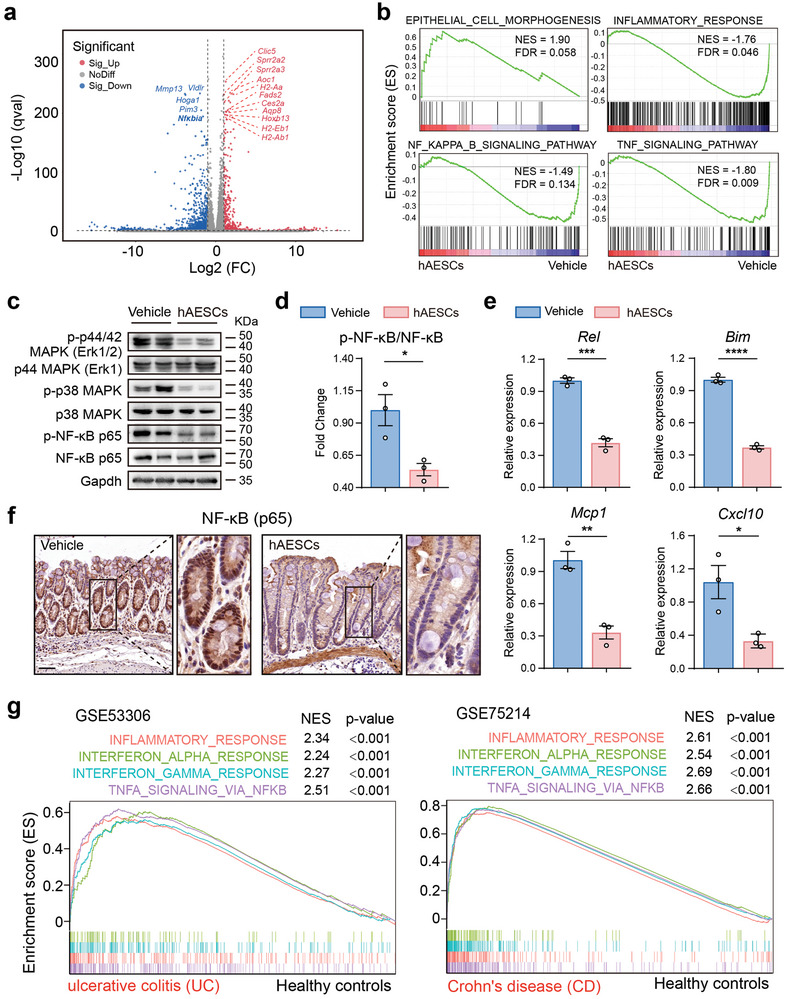
Protective effects of hAESCs in mice with DSS‐induced colitis via downregulation of the NF‐κB signaling pathway. a) Volcano plot showing the top differentially expressed genes in the colonic epithelia of hAESC‐treated and untreated mice with DSS‐induced colitis. Upregulated genes are shown in red, whereas downregulated genes are shown in blue. FC, fold change. b) Gene set enrichment analysis (GSEA) revealed the up‐ and down‐regulated pathways in hAESC‐treated mice. Genes are ranked along the X‐axis based on their expression levels, with upregulated genes on the left and downregulated genes on the right. Black vertical lines indicate the positions of individual genes in the gene set. Green line indicates the cumulative enrichment score along the Y‐axis. Positive normalized enrichment score (NES) indicates the enrichment of upregulated genes, whereas negative NES indicates the enrichment of downregulated genes. FDR, false discovery rate. c–e) Western blotting (c,d) and qRT‐PCR analysis (e) of the key factors in the tumor necrosis factor (TNF)/nuclear factor (NF)‐κB signaling pathway in the colonic epithelium. f) NF‐κB (p65) immunohistochemical staining of epithelial cells in the colonic mucosa of mice with DSS‐induced colitis with or without hAESC treatment. Scale bars = 100 µm. g) GSEA plot showing the gene set signatures in ulcerative colitis (UC) patient profiles in GSE53306 (left) and Crohn's disease (CD) patient profiles in GSE75214 (right). The corresponding *p* values are indicated. Data are represented as the mean ± SEM. Unpaired *t*‐test was used for analysis. n = 3. ****p < 0.0001, ***p < 0.001, and *p < 0.05.

To comprehensively investigate the effects of hAESCs on the colon after DSS‐induced injury, we conducted high‐throughput RNA sequencing of colonic epithelial samples. Transcriptomic analysis revealed the upregulation of 621 genes, including anti‐inflammatory genes (*Sprr2a*, *Fads2*, and *Hoxb13*) and MHC‐linked genes, in the hAESCs‐treated group compared to the control group. Previous studies have reported the crucial role of MHC‐linked genes in regulating inflammation and T‐cell responses under conditions of past injury.^[^ ^28^ ^]^ Conversely, 1474 genes were downregulated in the hAESCs‐treated group. Among them, *Mmp13*, which activates TNF signaling in epithelial and inflammatory cells, contributes to the escalation of local inflammation.^[^ ^29^ ^]^ Moreover, hAESCs treatment led to a downregulation of the downstream gene *Nfκbia* in the NF‐κB signaling pathway, indicating that hAESCs therapy can attenuate the activation of the NF‐κB signaling pathway induced by DSS exposure (**Figure** **4**a).

Gene set enrichment analysis unveiled an upregulation of gene sets associated with epithelial cell morphogenesis and a downregulation of gene sets related to inflammatory responses, defense response to bacterium, TNF signaling pathway, and NF‐κB signaling pathway in hAESCs treatment group (Figure 4b; Figure S4a, Supporting Information). Furthermore, western blot analysis demonstrated a significant reduction in the expression of proteins associated with TNF/NF‐κB pathway activation (phosphorylated p44, p38, and p65) in the hAESCs group compared to the control group (Figure 4c,d). RT‐qPCR analysis provided additional evidence for these findings, showing that hAESCs treatment led to decreased expression of genes associated with NF‐κB activation (*Rel*, *Bim*, *Mcp1*, and *Cxcl10*; Figure 4e). IHC staining of colon specimens from mice with DSS‐induced colitis revealed a decrease in NF‐κB translocation into the nucleus following hAESCs treatment of the colonic epithelial cells (Figure 4f).

The activation of TNF signaling has a potent effect on inducing cell death in intestinal epithelial cells, a process observed in the pathogenesis of various inflammatory diseases, particularly IBD.^[^ ^30^, ^31^ ^]^ As anticipated, hAESCs administration significantly suppressed the expression of genes associated with intestinal epithelial cell inflammatory injury (*Cxcl2, Hmox1*, *Reg3b*, *Reg3g*, and *Socs3*), while enhancing the expression of a proliferation‐associated gene (*Lgr5*) compared to the control group (Figure S4b, Supporting Information). IHC staining for cleaved Caspase‐3, a specific indicator of apoptosis, revealed a significant reduction in the number of positive cells within the colonic epithelium following hAESCs treatment (Figure S4c, Supporting Information), which was further validated by TdT‐mediated dUTP nick‐end labeling (TUNEL) assays (Figure S4d, Supporting Information). In addition, qRT‐PCR analysis of genes associated with apoptosis (*Fas*, *Bax*, and *Apaf1*) in colon epithelial cells supported these results (Figure S4e, Supporting Information). These findings suggest that hAESCs treatment may negatively regulate the TNF/NF‐κB signaling pathway, thereby alleviating inflammatory injury in the colon epithelium.

We further analyzed two clinical profiling datasets (GSE53306 and GSE75214) of IBD to validate the expression pattern of NFκB signaling in patients. The analysis demonstrated an increase in inflammatory response, IFNα response, IFNγ response, and TNFα signaling via NF‐κB in the colonic epithelium of both patients with ulcerative colitis and Crohn's disease compared to their normal counterparts (Figure 4g). These results further support our inference that negative regulation of the TNF/NF‐κB signaling pathway could be the underlying mechanism of hAESCs therapy for colitis. In summary, our study underscores the substantial influence of hAESCs on the colonic transcriptome, particularly in the modulation of crucial genes and pathways involved in NF‐κB signaling pathway.

### 2.5 hAESCs Exert Therapeutic Effects via Exosome Secretion

**Figure 5:**
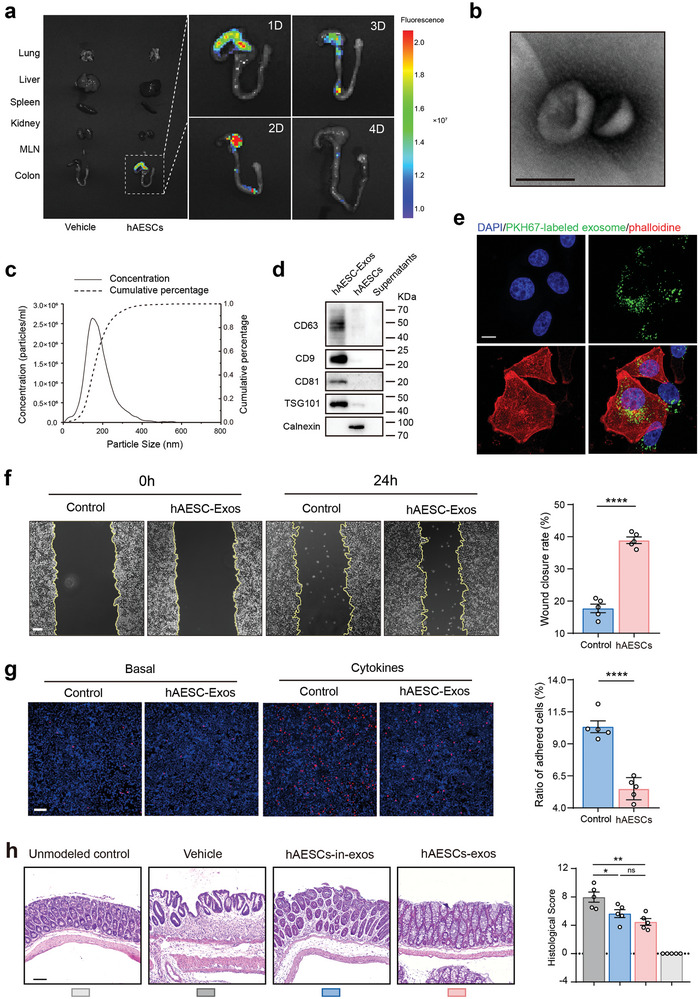
Characterization and effects of hAESC‐derived exosomes. a) NIR imaging and quantification of Dil dye‐labeled hAESCs following anal injections. NIR signals were captured from the five main organs (lungs, liver, spleen, kidneys, and large bowel) and corresponding MLNs daily post‐injection. b) Morphology of exosomes visualized via transmission electron microscopy. Scale bar = 100 nm. c) Particle size distribution of exosomes determined via nanoparticle tracking analysis (NTA). d) Western blotting analysis of exosome‐specific markers. e) Uptake of exosomes by FHC cells. FHC cells were incubated with PKH67‐labeled hAESC‐exos and imaged at 24 h via confocal microscopy. Scale bars = 10 µm. f) Evaluation of the wound healing ability of hAESC‐exo‐treated and untreated FHC cells under inflammatory stimuli at 24 h; quantification summary is shown on the right side. Scale bars = 100 µm. g) Assessment of adhesion between THP‐1 and FHC cells under inflammatory stimuli with or without pre‐incubation with hAESC‐exos; quantification summary is shown on the right side. Scale bars = 100 µm. h) H&E staining of colon tissue sections (left) and assessment of histological scores (right). Scale bar = 100 µm. Data are represented as the mean ± SEM. Unpaired *t*‐test or one‐way ANOVA followed by Tukey's post‐hoc test were used for analysis. n = 5. ****p < 0.0001, **p < 0.01, and *p < 0.05; ns, not significant (*p* > 0.05).

Successful engraftment of therapeutic cells at the site of injury is critical for determining the effectiveness of cytotherapy. To investigate the distribution of hAESCs in mice following anal injection, the non‐toxic near‐infrared (NIR) tracer DiI was used to label hAESCs. One day post injection, we evaluated the DiI signal intensity in various tissues and found that most hAESCs were trapped in the cecum. Notably, no cell migration to other organs (lungs, liver, spleen, and kidneys) and tissues (mesenteric lymph nodes) was observed. After two days, the DiI signal was initially detected in the colon, indicating that hAESCs gradually migrated distally along the colonic lumen over time. However, by the 4th day post‐injection, the NIR signal within the colon of mice treated with DSS significantly decreased and was nearly undetectable (**Figure** **5**a). Moreover, hAESCs stably infected with the luciferase gene were viable for at least three days during this period (Figure S5a, Supporting Information).

We further examined whether hAESCs, initially located in the colonic lumen, assimilate into the surrounding tissues. Histological analysis revealed no intact hAESCs within the epithelium or lamina propria. Instead, only faint NIR signals were observed (data not shown), indicating the presence of cell fragments or debris. Considering the affinity of the membrane dye DiI, we hypothesized that the therapeutic effects of hAESCs are predominantly mediated by the secretion of lipid membrane‐derived substances rather than by direct cellular interactions.

Cellular secretion is a vital process in cellular biology that involves the release of various bioactive molecules. One specific type of molecules is exosomes, which are a subtype of extracellular vesicles. Exosomes are typically 40–150 nm in diameter and play pivotal roles in tissue repair, regeneration, and intricate cell signaling.^[^ ^32^ ^]^ To investigate whether hAESCs exert their therapeutic effects via exosome‐mediated mechanisms, we first examined hAESC‐exos in vitro. Transmission electron microscopy and nanoparticle tracking analysis (NTA) demonstrated that hAESCs‐exos possessed a distinct hollow spherical morphology (Figure 5b), with a size distribution ranging from 40 to 150 nm (Figure 5c). In addition, the presence of exosome‐specific proteins (CD63, CD9, CD81, and TSG101) was identified by western blotting, and the absence of Calnexin, a marker of the endoplasmic reticulum, further verified the purity of hAESCs‐exos (Figure 5d). Subsequently, hAESCs‐exos were labeled with fluorescent dye PKH67 and incubated with FHC cells. Fluorescence microscopy demonstrated efficient uptake of hAESCs‐exos by epithelial cells (Figure 5e). These findings contribute to our understanding of the potential therapeutic effects of exosomes in hAESCs.

Based on the unique characteristics of hAESCs‐exos, we further investigated their effects on epithelial cell proliferation and adhesion under inflammatory conditions. Our findings demonstrated that incubation with hAESCs‐exos significantly promoted the closure of epithelial wounds (Figure 5f) and effectively inhibits THP1‐epithelium adhesion (Figure 5g) in the presence of TNF stimulation. These observations highlight the promising role of hAESCs‐exos in the modulation of cellular responses to inflammation.

To verify the therapeutic effects of hAESC‐derived exosomes, we first confirmed the release of exosomes from hAESCs encapsulated in the RGD hydrogel using a transwell assay in vitro (Figure S5b,c, Supporting Information). Quantitative analysis of exosomes secreted by hAESCs was performed using NTA, revealing ≈3.44 × 10^10^ ± 3.15 × 10^9^ particles released from the RGD hydrogel encapsulating 1 × 10^6^ hAESCs (Figure S5d,e, Supporting Information).

In vivo, hAESC‐exos and hAESCs incubated with an exosomal inhibitor were hydrogel‐encapsulated and administered to DSS‐induced colitis model mice (Figure S6a, Supporting Information). Indeed, treatment with hAESCs‐exos inhibited colonic shortening and body weight loss during the recovery phase in mice with DSS‐induced colitis (Figure S6b–d, Supporting Information). Histological analyses revealed the significant reduction in colonic injury and inflammation following hAESCs‐exos administration (Figure 5h). Immunofluorescence and RT‐qPCR analyses revealed the significant increase in epithelial cell proliferation (Figure S6e–g, Supporting Information) and decrease in inflammatory cell infiltration (Figure S6h, Supporting Information) after hAESC‐exos treatment. Moreover, intestinal barrier repair was enhanced in hAESCs‐exos‐treated colitis mice compared to that in the vehicle control mice (Figure S6i,j, Supporting Information). In contrast, effectiveness of hAESCs treatment was partially reduced when hAESCs were treated with an exosomal inhibitor, even when administered at the same cell number.

In summary, our study highlights the therapeutic potential of hAESCs in modulating inflammatory responses and enhancing regeneration through exosome secretion. This discovery underscores the significance of exosome‐mediated mechanisms in the observed therapeutic effects of hAESCs and offers new insights into their future therapeutic applications.

### 2.6 hAESCs‐Derived Exosomal miR‐23a‐3p Inhibits NF‐κB Signaling Activation by Directly Targeting TNFR1

**Figure 6:**
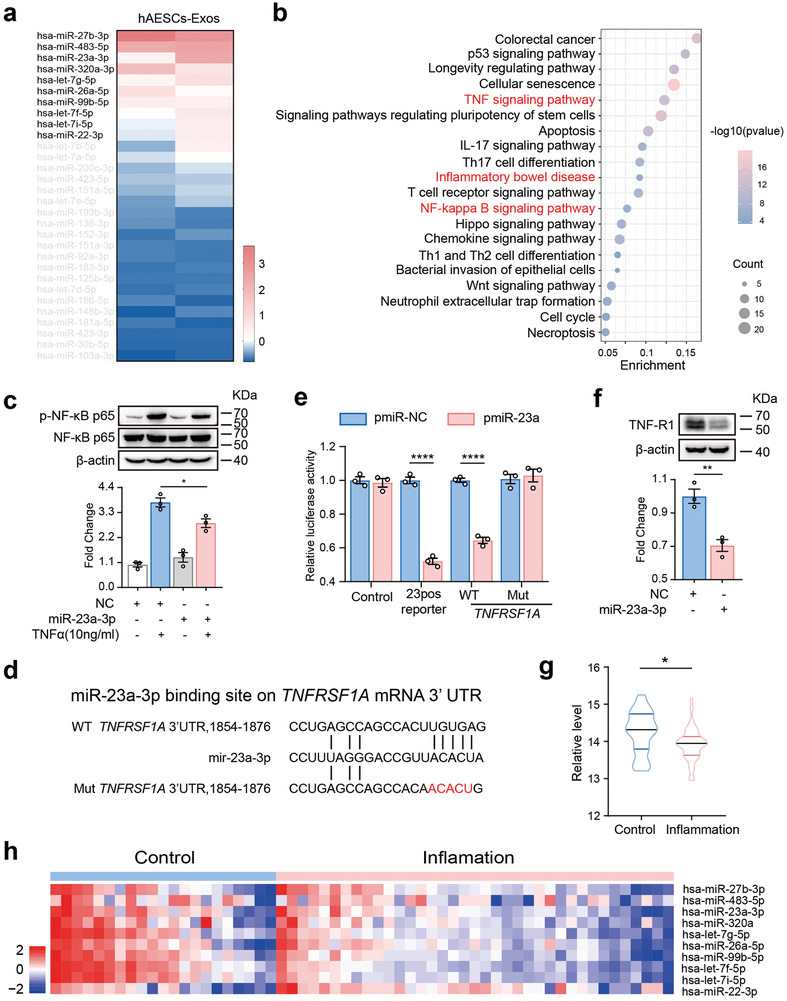
Inhibition of NF‐κB signaling by hAESC‐derived exosomal microRNA (miR)‐23a‐3p via direct targeting of tumor necrosis factor receptor 1 (*TNFR1*). a) Expression levels of miRNAs in hAESC‐exos (n = 2) determined via small RNA sequencing. b) Bubble chart showing the enrichment analysis of target genes of miRNAs enriched in hAESC‐exos via Kyoto Encyclopedia of Genes and Genomes (KEGG) pathway analysis. c) Western blotting analysis of the key factors in the NF‐κB signaling pathway in Caco‐2 cells. Caco‐2 cells transfected with or without miR‐23a‐3p mimic were treated with TNF‐α (10 ng mL^−1^) for 15 min. Protein levels were normalized to those of NF‐κB p65 and NC group. d) Illustration of miR‐23a‐3p‐binding sites on the 3′‐UTR of *TNFRSF1A* mRNA. e) Dual luciferase assay showing the miR‐23a‐3p‐mediated repression of wild‐type and mutant 3′‐UTR of *TNFRSF1A* mRNA. Luciferase activity was normalized to that of the NC group. f) Western blotting analysis of TNF‐R1 protein levels in FHC cells transfected with the miR‐23a‐3p mimic or NC. Protein levels were normalized to those of β‐actin and NC group. g) Violin plots showing the median, interquartile range, 95% confidence intervals, and frequency of expression of miR‐23a‐3p in each cluster. h) Heat map showing the hAESC‐exosomal miRNA expression levels in the gene sets of patients with IBD. Colors on the heat map reflect the gene expression values normalized based on the mean expression of miRNA across all samples. Blue indicates gene downregulation, whereas red indicates gene upregulation in the tissues. Data are represented as the mean ± SEM. Unpaired *t*‐test was used for analysis. n = 3. ****p < 0.0001 and *p < 0.05.

Exosomes contain various molecules, including proteins, lipids, DNA, and RNA.^[^ ^33^ ^]^ Among these, miRNAs are the most bioactive molecules that have attracted attention because of their regulatory roles in gene expression. We previously demonstrated the crucial roles of miRNAs in intestinal epithelial regeneration_._^[^ ^33^, ^34^ ^]^ To further understand the mechanisms underlying the inhibitory effect of hAESCs‐exos on the NF‐κB signaling pathway, Illumina HiSeq sequencing was used to assess the abundance of specific miRNAs in hAESCs‐exos. Among the 572 miRNAs analyzed, miR‐27b‐3p, miR‐483‐5p, miR‐23a‐3p, miR‐320a‐3p, let‐7g‐5p, miR‐26a‐5p, miR‐99b‐5p, let‐7f‐5p, let‐7i‐5p, and miR‐22‐3p exhibited stable and relatively high expression levels in hAESCs‐exos (**Figure** **6**a). Subsequently, we used the bioinformatics tools, miRDB and TargetScan, to predict the target genes of these miRNAs. Target genes were further assessed via Kyoto Encyclopedia of Genes and Genomes pathway enrichment analysis. The genes were significantly enriched in pathways associated with inflammation, particularly the TNF/NF‐κB signaling pathway (Figure 6b). To identify the predominant miRNA responsible for suppressing NF‐κB activation during inflammation, Caco2 human epithelial cell line were transfected with miRNA mimics and stimulated with TNF to activate NF‐κB with p65 phosphorylation. Western blotting revealed that miR‐23a‐3p overexpression partially rescued the activation of NF‐κB signaling pathway (Figure 6c).

Next, miRDB and TargetScan were used to predict potential miRNA targets among the key components of the TNF/NF‐κB signaling pathway to elucidate the mechanism by which miR‐23a‐3p regulates the NF‐κB signaling pathway. We discovered a binding region for miR‐23a‐3p within the 3′‐UTR of the TNF receptor, *TNFR1* mRNA (Figure 6d). Luciferase activity assay demonstrated that miR‐23a‐3p overexpression significantly reduced the luciferase reporter activity in the wild‐type 3′‐UTR of human *TNFR1*, while no significant change was observed in the mutant reporter (Figure 6e). Furthermore, miR‐23a‐3p overexpression led to a decrease in TNFR1 protein levels in Caco2 cells (Figure 6f). These findings strongly indicate that *TNFR1* is a direct target of miR‐23a‐3p.

Bioinformatic analysis of public databases revealed significantly lower miR‐23a‐3p levels in the inflamed mucosa than in the normal mucosa of patients with IBD (Figure 6g), suggesting the potential role of miR‐23a‐3p in IBD pathogenesis. The top 10 miRNAs in hAESC‐derived exosomes were also significantly downregulated in the inflamed samples, indicating their involvement in the disease process (Figure 6h). In summary, these findings suggest that miR‐23a‐3p in hAESCs‐exos binds to the 3′‐UTR of *TNFR1* mRNA, thereby inhibiting its translation and suppressing the activation of NF‐κB signaling pathway.

### 2.7 hAESCs‐Derived Exosomal miR‐23a‐3p Plays Key Roles in Ameliorating DSS‐Induced Colitis

**Figure 7:**
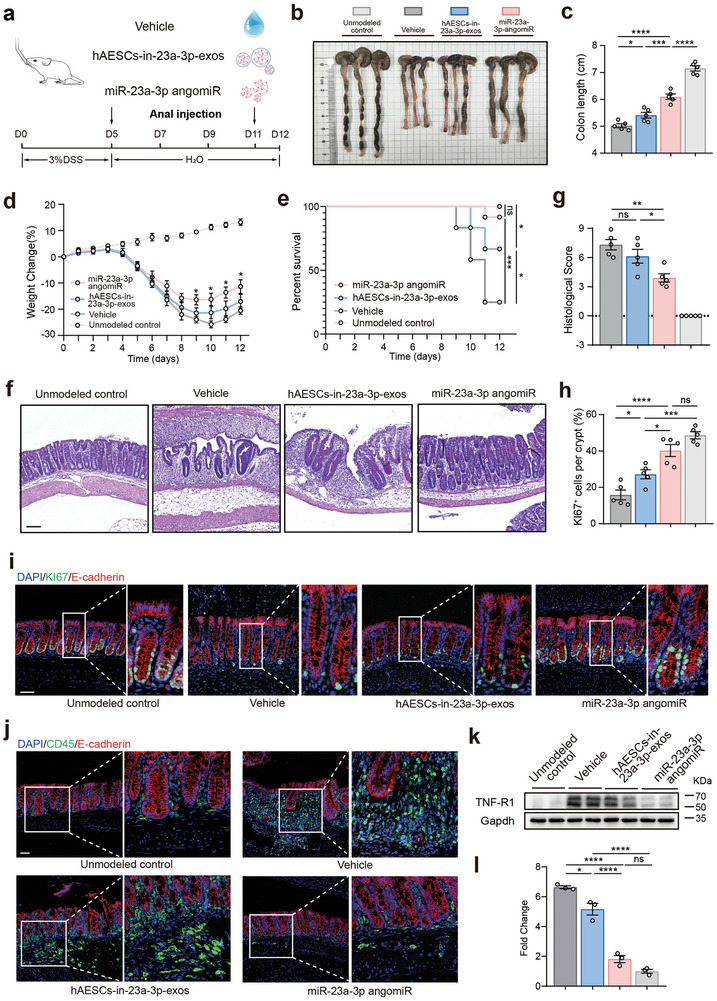
Attenuation of hAESC‐exo‐mediated amelioration of DSS‐induced colitis by inhibiting exosomal miR‐23a‐3p. a) Schematic representation of the injection protocol for mice with DSS‐induced colitis. b, c) Assessment of colon morphology (b) and colon length in each group (c). d, e) Changes in body weights (d) and survival (e) of mice during DSS‐induced colitis induction and recovery. f, g) H&E staining of colon sections (f) and evaluation of histological scores (g) in mice on day 12. Scale bars = 100 µm. h, i) Evaluation of the regenerated epithelial cells in the colonic mucosa of mice with DSS‐induced colitis via Ki67 immunofluorescence staining (i). Quantification summary is shown in (h). Scale bars = 50 µm. j) Assessment of inflammatory cell infiltration in the colonic mucosa of mice with DSS‐induced colitis via CD45 (green) immunofluorescence staining. Scale bars = 50 µm. k, l) Western blotting analysis of TNF‐R1 protein levels in the colonic epithelia of mice with DSS‐induced colitis (k). Protein levels were normalized to those of glyceraldehyde‐3‐phosphate dehydrogenase (Gapdh) (l). Data are represented as the mean ± SEM. One‐way ANOVA followed by Tukey's post‐hoc test was used for analysis. n = 5. ****p < 0.0001, ***p < 0.001, **p < 0.01, and *p < 0.05; ns, not significant (*p* > 0.05).

To assess the importance of exosomal miR23a‐3p in the therapeutic efficacy of hAESCs, an miR‐23a‐3p inhibitor was used to suppress hAESCs‐derived exosomal miR‐23a‐3p (hAESCs‐in‐miR‐23a‐3p‐exos). Briefly, hydrogel‐encapsulated hAESCs‐in‐miR‐23a‐3p‐exos or miR‐23a‐3p angomiR were administered via anal injection to mice with DSS‐induced colitis every other day, with the control group only receiving the hydrogel (**Figure** **7**a). Compared to the mice receiving hAESCs‐in‐miR‐23a‐3p‐exos, those receiving miR‐23a‐3p angomiR exhibited significantly reduced colon length (Figure 7b,c), rapid body weight recovery following DSS withdrawal (Figure 7d), and enhanced survival (Figure 7e). In addition, miR‐23a‐3p angomiR groups showed improved epithelial structures (Figure 7f) and histological scores (Figure 7g). Moreover, significantly increased epithelial cell proliferation (Figure 7h,i) and decreased inflammatory cell infiltration (Figure 7j) were observed in the miR‐23a‐3p angomiR group compared to those in the hAESCs‐in‐miR‐23a‐3p‐exos group. miR‐23a‐3p angomiR‐treated mice exhibited lower TNFR1 levels than the hAESCs‐in‐miR‐23a‐3p‐exos‐treated mice during the recovery phase (Figure 7k,l). Interestingly, hAESCs treatment further moderately improved the therapeutic effects of Infliximab (a TNF inhibitor), suggesting that TNF signaling is the major target of hAESCs therapy. However, the therapeutic effects of hAESCs are multifaceted and may involve some other molecular pathways in addition to TNF signaling (Figure S7, Supporting Information).

These findings suggest the crucial role of miR‐23a‐3p in exosomes for the therapeutic effects of hAESCs against acute colitis.

## 3 Discussion

Impairment of the intestinal barrier, which facilitates the translocation of microorganisms and antigens across the intestinal epithelium and subsequent uncontrolled immune activation, is a critical characteristic of IBD.^[^ ^35^, ^36^ ^]^ Although the restoration of barrier function is the main therapeutic objective in IBD,^[^ ^35^, ^37^, ^38^, ^39^ ^]^ effective therapeutic methods remain scarce. In this study, we found that hAESCs enhanced mucosal healing, a functional indicator of barrier restoration, in mice with DSS‐induced colitis during the recovery phase. This effect was mediated by the secreted exosomes with significantly elevated miR‐23a‐3p levels. Further mechanistic investigations revealed that miR‐23a‐3p targeted the 3′‐UTR of *TNFR1* mRNA to post‐transcriptionally regulate the TNFR1 protein levels and subsequently suppress the NF‐κB pathway.

Cell‐based therapies show great potential for tissue regeneration. However, their in vivo use is limited by cell survival issues in a hostile microenvironment. Stem cells in single‐cell suspensions often show poor survival because of anoikis, a phenomenon that occurs without cell attachment.^[^ ^40^, ^41^ ^]^ To enhance cell survival and regenerative capacity upon infusion into the colonic lumen, we utilized tunable RGD hydrogels to encapsulate hAESCs, providing a protective shield against unfavorable environments. To determine the optimal growth environment for hAESCs, we adjusted the concentration of hydrogels by diluting them at various ratios. Ultimately, 5‐times dilution of RGD hydrogels was identified as the optimal environment for hAESCs. In addition, NIR fluorescence imaging revealed that hAESCs persisted within the colon lumen for up to four days without engraftment, suggesting that they functioned by acting on the colonic epithelial cells rather than by replacing the damaged tissues. Furthermore, hydrogel accumulated in the colonic lumen and did not translocate to other organs, demonstrating the safety and efficacy of the hydrogel‐encapsulated hAESCs composites.

Many studies have highlighted the tissue repair capacity of hAESCs primarily via immunomodulatory mechanisms. For instance, hAESCs enhance survival and mitigate renal injury by promoting M2 macrophage polarization and suppressing systemic inflammation in a mouse model of ischemia/reperfusion injury‐induced acute kidney injury.^[^ ^42^ ^]^ Moreover, hAESCs treatment induces the maturation of non‐Tregs into FoxP3‐expressing Tregs and shifts macrophage polarization from pro‐inflammatory M1 to anti‐inflammatory M2 phase, thereby alleviating lung inflammation and fibrosis in the bleomycin‐induced Rag1^−/−^ mouse model.^[^ ^43^ ^]^ However, in an animal model of premature ovarian failure/insufficiency, hAESCs treatment exhibited a distinct mechanism by inhibiting chemotherapy‐induced apoptosis in primary granulosa‐lutein cells rather than by modulating the immune responses.^[^ ^44^ ^]^ In the present study, Dil‐labeled hAESCs assembled in the colonic lumen without entering the lamina propria of the colon. Interestingly, hAESCs‐derived exosomes were efficiently internalized by the intestinal epithelial cells, subsequently promoting mucosal healing and thus improving the intestinal epithelial barrier dysfunction. To the best of our knowledge, this study is the first to demonstrate that hAESCs exert therapeutic effects directly on intestinal epithelial cells, irrespective of the inflammation condition. This provides a novel approach to manage and treat IBD and reduce the dependence on traditional immunosuppressive therapies.

Initially, therapeutic potential of stem cells was mainly associated with tissue regeneration by engraftment at the site of injury and replacement of damaged cells. However, subsequent studies revealed that stem cell engraftment at the injury site is very rare.^[^ ^45^ ^]^ Our results also confirm the absence of hAESCs integration into the intestinal tissue after injection. Increasing evidence in regenerative medicine supports the hypothesis that stem cells primarily exert their therapeutic effects via paracrine mechanisms rather than direct repopulation. This paracrine activity often involves the secretion of exosomes that play crucial roles in cell–cell communication by transferring bioactive molecules from the donor to recipient cells.^[^ ^46^, ^47^ ^]^ A previous study reported the potential of hAESCs‐derived exosomes to mitigate lung injury and promote the proliferation of bronchoalveolar stem cells, partly by enriching the contents of functional proteins and miRNAs after bleomycin challenge.^[^ ^48^ ^]^ Additionally, hAESCs‐derived exosomes restore the ovarian function in cases of chemotherapy‐induced ovarian failure by delivering miRNAs to inhibit apoptosis.^[^ ^49^ ^]^ Consistently, our study showed that the introduction of hAESCs‐derived exosomes replicated the protective effects of hAESCs in the DSS‐induced colitis model. This observation further supports the notion that hAESCs primarily confer their therapeutic effects via exosome secretion.

miRNAs are short endogenous non‐coding RNAs that regulate gene expression by binding to the 3′‐UTR of mRNAs, resulting in mRNA degradation or repression of translation of the target genes. In this study, our investigations revealed high expression levels of several miRNAs, including miR‐27b‐3p, miR‐483‐5p, miR‐23a‐3p, miR‐320a‐3p, and let‐7g‐5p, in hAESCs‐exos. Importantly, these exosomal miRNAs predominantly targeted the genes associated with IBD, which were enriched in the Il‐17, Th17 cell differentiation, TNF, NF‐κB, bacterial invasion of epithelial cells, Hippo, and Wnt signaling pathways. These pathways are closely related to the immune response and intestinal epithelial function. These findings suggest that hAESCs‐exos perform their biological functions by delivering specific miRNAs to the target cells involved in these pathways.

TNF, a key mediator of inflammatory responses implicated in the pathogenesis of various human diseases, including IBD, becomes pathogenic on aberrant/excess activation. TNF activates the downstream signals via two cell surface receptors, TNF‐R1 and TNF‐R2, with TNF‐R1 primarily initiating biological activities that lead to the activation of NF‐κB and JNK pathways.^[^ ^50^ ^]^ In our study, administration of hAESCs resulted in the downregulation of TNF/NF‐κB signaling, with hAESCs‐exo‐derived miR‐23a‐3p directly downregulating *TNFR1* levels. The antagonistic effects of miR‐23a‐3p inhibitor and angomiR on hAESCs‐mediated colitis recovery suggest the exosomal miR‐23a–TNFR1–NF‐κB axis as the central mechanism responsible for hAESCs therapeutic effects against colitis. Therefore, administration of hAESCs with miR‐23a‐3p or miR‐23a‐3p‐enriched hAESCs‐exos is a potential therapeutic strategy for IBD in clinic.

The main limitation of this study is the sole use of the DSS colitis model, which, although widely used in epithelial repair studies, does not fully recapitulate the complexity of human IBD. Future studies should use more complex models, such as genetically modified mice or patient‐derived organoids, for comprehensive evaluation of the therapeutic potential of hAESCs. Furthermore, clinical trials are essential to confirm the efficacy and safety of hAESCs for patients with IBD.

## 4 Conclusion

In conclusion, this study highlights the therapeutic potential of hAESCs and their exosomes to for mucosal healing and intestinal epithelial restoration during the recovery phase of colitis. The crucial roles of miR‐23a‐3p and its regulation of the TNFR1–NF‐κB pathway indicate promising avenues for future investigations, to optimize the treatment strategies for IBD and associated diseases and improve the patient outcomes.

## 5 Experimental Section

### hAESCs Isolation and Culture

Human amniotic membranes were obtained after cesarean sections from healthy mothers with their written informed consent. This study was approved by the Institutional Patients and Ethics Committee of the Second Affiliated Hospital of Zhejiang University School of Medicine (ethics code: 2020–799). All donors tested negative for hepatitis A, B, C, and D, human immunodeficiency virus‐I, and *Treponema pallidum* antibodies. Subsequently, hAESCs were isolated and cultured as previously described.^[^ ^51^ ^]^ Briefly, the amniotic membrane was separated from the placental chorion, washed with Hank's balanced salt solution, and incubated with 0.25% trypsin at 37 °C for 20 min. Following centrifugation at 1000 rpm for 4 min at 25 °C, harvested cells were quantified and cultured in a complete medium optimized for the selective growth of hAESCs in vitro, which suppressed the growth of other amniotic membrane and hematopoietic cells. The medium used was the Dulbecco's modified Eagle's medium/nutrient mixture F12 supplemented with 10% KnockOut serum replacement, 2 mM L‐glutamine, 1% nonessential amino acids, 55 µM 2‐mercaptoethanol, 1 mM sodium pyruvate, and 100 U mL^−1^ penicillin/streptomycin (all from Thermo Fisher Scientific, Waltham, USA). The medium was also supplemented with 10 ng mL^−1^ human epidermal growth factor (Peprotech, NJ, USA) and refreshed every 2–3 d.

### Animals and Treatments

Male and female C57BL/6 mice aged between 8–12 weeks were obtained from Shanghai SLAC Laboratory Animal Co., Ltd. and housed in a pathogen‐free facility at the Laboratory Animal Center of Zhejiang University. The mice were kept in rooms with controlled temperature and lighting and provided ad libitum access to a standard chow diet and water. All animal experiments were approved by the Zhejiang University Institutional Animal Care and Research Committee (approval number ZJU20230403) and conducted according to NIH guidelines for the ethical treatment of animals. To induce colitis, the mice were administered water containing 3% DSS (YEASEN, Shanghai, China) for five days, followed by the administration of normal drinking water. On days 6 and 9, the mice received anal injections of hAESCs (1 × 10^6^ cells) suspended in 200 µL of high‐concentration RGD hydrogels (1:5 ratio; TWG003; The Well bioscience, NJ, USA). Anal injections were administered every other day from days 5 to 11 using a flexible catheter 4 cm in length and 2 mm in diameter.^[^ ^52^ ^]^ The control group was challenged with DSS and received diluted high‐concentration RGD hydrogel without hAESCs or hAESCs‐exos.

### Assessment of Colitis Severity and Intestinal Permeability in Mice

Body weights of mice were recorded at the same time daily. Fresh feces were collected from the animals for occult blood testing using a fecal occult blood kit (Baso, Zhuhai, China), following the manufacturer's instructions. Disease activity index was determined using a scoring system considering body weight loss, stool consistency, and rectal bleeding.^[^ ^53^ ^]^ Intestinal permeability was assessed via the oral administration of FITC‐labeled dextran, as previously described.^[^ ^54^ ^]^ On day 12, all mice were euthanized and the entire colon, from the cecum to the anus, was excised. Then, colon length was measured to determine the colon length‐to‐body weight ratio.

### NIR Dye Labeling and Fluorescent Imaging

The NIR fluorescent dye, 1,1′‐dioctadecyl‐3,3,3′,3′‐tetramethylindocarbocyanine perchlorate (DiIC_18_(3); Beyotime, Shanghai, China), was used to label the cells, according to the manufacturer's instructions. Briefly, adherent cells were exposed to 5‐µM Dil solution for 15 min at 37 °C. Subsequently, the labeled cells were washed twice with warm fresh medium for the complete removal of any unbound dye. All fluorescence imaging procedures were performed using a small‐animal imaging system (IVIS Spectrum; PerkinElmer, Sollentuna, Sweden). Then, fluorescence intensity was quantified and analyzed using the Living Image Software (version 4.2; PerkinElmer).

### Histopathological Analysis

Colon tissues were fixed with 4% paraformaldehyde (pH 7.4) and subjected to gradual dehydration. The tissues were subsequently embedded in paraffin, and 5‐µm sections were prepared and stained with hematoxylin and eosin. Histological scores were determined from 3–5 independent fields using a combined scoring system considering epithelial damage and inflammatory cell infiltration, as previously reported.^[^ ^55^ ^]^

### IHC and Immunofluorescence Assays

For IHC, deparaffinized sections were subjected to endogenous peroxidase quenching, antigen retrieval, and subsequent blocking. Then, the slices were incubated overnight at 4 °C with primary antibodies against cleaved caspase‐3 mAb (1:2000; 9664) NF‐κB p65 mAb (1:1000; 8242; both from Cell Signaling Technology, MA, USA). After incubation with biotinylated secondary antibodies for 1 h at room temperature, immunosignals were visualized using the DAB kit (NeoBioscience, Beijing, China). For immunofluorescence assay, the deparaffinized sections were subjected to antigen retrieval, blocking, and incubated with primary antibodies against E cadherin (1:1000; ab231303), Ki67 (1:1000; ab15580), Muc2 (1:500; ab272692) (all from Abcam, Cambridge, UK), and CD45 (1:100; 70 257; Cell Signaling Technology, MA, USA) overnight at 4 °C. After washing with phosphate‐buffered saline (PBS), the sections were incubated with DyLight 488‐conjugated goat anti‐rabbit IgG (1:400; ab96899; Abcam, Cambridge, UK) and Alexa Fluor 594‐conjugated donkey anti‐mouse IgG (1:1000; R37115; Invitrogen, USA) secondary antibodies at room temperature for 1 h. After 4′,6‐diamidino‐2‐phenylindole staining (1:500; Solarbio, Beijing, China), the sections were sealed with the antifade mounting medium (FDbio, Hangzhou, China) and observed using a FV3000 confocal laser scanning microscope (Olympus, Japan).

### Alcian Blue and Periodic Acid‐Schiff Assays

Paraffin‐embedded sections were analyzed using the Alcian Blue & Nuclear Fast Red and Periodic Acid‐Schiff Staining Kits (Beyotime, Shanghai, China) for in situ MUC detection, according to the manufacturer's instructions.

### TUNEL Assay

Paraffin‐embedded sections were analyzed using the TUNEL Apoptosis Detection Kit (YEASEN, Shanghai, China) for in situ apoptosis detection, according to the manufacturer's instructions. DNase I‐treated samples were used as positive controls.

### Exosome Isolation and Identification

After reaching 70% confluency, the culture medium was replaced, and hAESCs were incubated at 37 °C with 5% CO_2_ for 48 h. Then, centrifugation was performed at 2000 × *g* for 10 min to remove the debris and dead cells, followed by a second centrifugation at 10 000 × *g* for 30 min at 4 °C to further eliminate any remaining debris. The resulting supernatant was processed via ultracentrifugation at 100 000 × *g* for 70 min at 4 °C to isolate the exosomes. The pelleted exosomes were washed with PBS and subjected to ultracentrifugation at 100 000 × *g* for 90 min at 4 °C. Finally, the exosome‐containing pellets were resuspended in PBS.

Exosome morphology was examined using transmission electron microscopy, and the particle size distribution was analyzed via NanoSight analysis (NanoSight Ltd., MA, USA). The presence of specific exosomal proteins was confirmed via western blotting using antibodies against CD66, CD9, CD81, TSG101, and calnexin^[^ ^56^ ^]^ (all from Abcam, Cambridge, UK). Furthermore, uptake of exosomes was visualized using the PKH67 Green Fluorescent membrane linker dye (Solarbio, Beijing, China), following the manufacturer's instructions.

### Cell Culture and Treatments

FHC cell line and complete medium were obtained from Meisen CTCC. THP‐1 and Caco2 cell lines were cultured in the Roswell Park Memorial Institute‐1640 (Gibco, USA) supplemented with 10% FBS (Vistech, USA) and 100 U mL^−1^ penicillin/streptomycin (Gibco) at 37 °C in a 5% CO_2_ atmosphere. To establish the cellular inflammatory model, FHC cells were pre‐incubated with or without hAESC‐exos for two days, followed by stimulation with 25 ng mL^−1^ TNF‐α (Sigma‐Aldrich, USA) for 24 h. Wound healing assays were conducted by creating a scratch in the cell monolayer using a 10‐µL pipette tip, followed by washing with PBS. Images were captured using a Nikon microscope at specific time points, and the wound areas were measured using the ImageJ software. For adhesion assays, THP‐1 cells were stained with calcein (Invitrogen, USA) and co‐cultured with FHC for 30 min. Non‐adherent cells were gently washed away, and microphotographs were taken by a fluorescence microscope (Nikon, Japan). The cell nuclei were stained with 4′,6‐diamidino‐2‐phenylindole (1:500; Solarbio, Beijing, China). At least three biological replicates were used for analysis.

### miRNA Transfection

The miR‐23a‐3p‐inhibitor, miR‐23a‐3p agomiR, and negative control (GenePharma, Shanghai, China) were transfected into the FHC cells, Caco2 cells, and hAESCs at a final concentration of 100 nM using the Hieff Trans in vitro siRNA/miRNA Transfection Reagent (YEASEN, Shanghai, China). All miR‐23a‐3p‐inhibitor/agomiR sequences are listed in the Table S1 (Supporting Information).

### Dual Luciferase Assay

Next, 293T cells were co‐transfected with the miR‐23a‐3p expression plasmid or empty vector, along with a firefly luciferase reporter plasmid containing the wild‐type or mutant miR‐23a‐3p‐binding sites on the 3′‐UTR of *TNFR1* mRNA and the Renilla luciferase internal control plasmid. After 48 h, the cells were lysed and firefly and Renilla luciferase activities were measured using the dual‐luciferase reporter assay. At least three biological replicates were used for analysis.

### RNA Isolation and Quantitative Real‐Time PCR

Total RNA was extracted from the colonic epithelium of mice using the TRIzol reagent (Invitrogen, USA). Reverse transcription was performed using the Evo M‐MLV RT Premix for qPCR (Accurate Biology, Hunan, China), and mRNA levels were quantified via RT‐qPCR using the SYBR Green Premix Pro Taq HS qPCR Kit (Accurate Biology, Hunan, China). Relative expression levels were determined using the 2^−ΔΔCT^ method. All gene‐specific primers used in this study are listed in the Table S1 (Supporting Information).

### High‐Throughput Sequencing Analysis

For RNA‐sequencing, RNA libraries were prepared using the Illumina Novaseq 6000 platform. Data analyses, including pathway analysis, generation of volcano maps, and gene set enrichment analysis, were performed using OmicStudio tools (http://www.omicstudio.cn/tool) in the R programming language (https://www.r‐project.org. For miRNA sequencing, total RNA was extracted from exosomes using the Total Exosome RNA and Protein Isolation Kit (Invitrogen, USA). The quantity and quality of small RNA in the total RNA were assessed by LC Bio Technology Co., Ltd. (Hangzhou, China). Additionally, small RNA libraries were constructed and sequenced by the same company.

### Western Blotting Analysis

Caco‐2 cells were lysed using a lysis buffer containing 0.1% Triton X‐100, 50 mM Tris‐HCl (pH 7.5), 150 mM NaCl, 1 M EGTA, and 1 M EDTA and supplemented with a protease and phosphatase inhibitor cocktail (YEASEN, Shanghai, China). Colonic tissues were lysed using the radioimmunoprecipitation assay lysis buffer (Strong; Yoche, Shanghai, China). Equal amounts of protein samples were separated via 12% sodium dodecyl sulfate‐polyacrylamide gel electrophoresis, transferred to polyvinylidene difluoride membranes, and incubated with primary antibodies against CD63, CD9, CD81, TSG101, and calnexin (ab275018; Exosome Panel; Abcam, Cambridge, UK), extracellular signal‐regulated kinase (ERK)‐1 (sc‐94), p38 alpha/beta mitogen‐activated protein kinase (MAPK sc‐7972), p‐p38 MAPK (sc‐166182) (all from Santa Cruz Biotechnology, CA, USA), phosphor‐p38 MAPK (4511), and phosphor‐p44/42 MAPK (Erk1/2; 9101) (both from Cell Signaling Technology, MA, USA) overnight at 4 °C. The membranes were subsequently incubated with the appropriate horseradish peroxidase‐conjugated secondary antibodies for 1 h at room temperature. Chemiluminescence was detected using an ECL kit (FDbio, Hangzhou, China), and chemiluminescent signals were visualized using the Tanon 5200 Multisystem (Tanon, Shanghai, China). Then, signal intensity of each protein band was quantified using the ImageJ software and normalized to that of β‐actin (1:10000; AC004; Abclonal, Wuhan, China). Three biological replicates were used for analysis.

### Statistical Analyses

Data analysis was performed using GraphPad Prism version 8 (GraphPad Software, USA). Results are represented as the mean ± standard error of the mean. Images were processed using the ImageJ, Photoshop CS, and Illustrator CS software programs. Statistical significance was determined using the Student's *t*‐test or one‐way analysis of variance followed by Tukey's post‐hoc test, as appropriate, with significance levels set at *p < 0.05, **p < 0.01, ***p < 0.001, and ****p < 0.0001. Information of sample size (n number) for statistical analyses was indicated in correlated figure legends.

## Conflict of Interest

The authors declare no conflicts of interest.

## Author Contributions

Y.K., X.W., and L.Y. designed and supervised the study. Y.K., Y.Z., J.L., R.R., Y.J., Y.W., C.Q., J.Z., Z.Y., T.J., J.H., X.R., and S.L. performed the experiments. Y.K., J.L., C.Q., and X.W. analyzed and interpreted the data. Y.K., X.W., and L.Y. drafted and revised the manuscript. X.W. and L.Y. approved the manuscript for submission.

## Supporting information



Supporting Information

Supplemental Table 1

## Data Availability

The data that support the findings of this study are available from the corresponding author upon reasonable request.
