# Supplementary material for: Human Amniotic Epithelial Stem Cells Promote Colonic Recovery in Experimental Colitis via Exosomal MiR‐23a–TNFR1–NF‐κB Signaling
Source: Adv Sci (Weinh). 2024 Oct 8;11(44):2401429. doi: 10.1002/advs.202401429 (PMC11600273; doi:10.1002/advs.202401429)
Supplement: Supplementary file 1 — Supporting Information [file ADVS-11-2401429-s001.pdf]

ADVANCED SCIENCE
Open Access

Supporting Information

for *Adv. Sci.*, DOI 10.1002/advs.202401429

Human Amniotic Epithelial Stem Cells Promote Colonic Recovery in Experimental Colitis via Exosomal MiR-23a–TNFR1–NF-κB Signaling

Yaohui Kou, Jinying Li, Yingyi Zhu, Jia Liu, Ruizhe Ren, Yuanqing Jiang, Yunyun Wang, Chen Qiu, Jiayi Zhou, Zhuoheng Yang, Tuoying Jiang, Jianan Huang, Xiangyi Ren, Shiguang Li, Cong Qiu, Xiyang Wei and Luyang Yu**

Human Amniotic Epithelial Stem Cells Promote Colonic Recovery in Experimental Colitis via Exosomal MiR-23a–TNFR1–NF-κB Signaling

Supplementary Materials

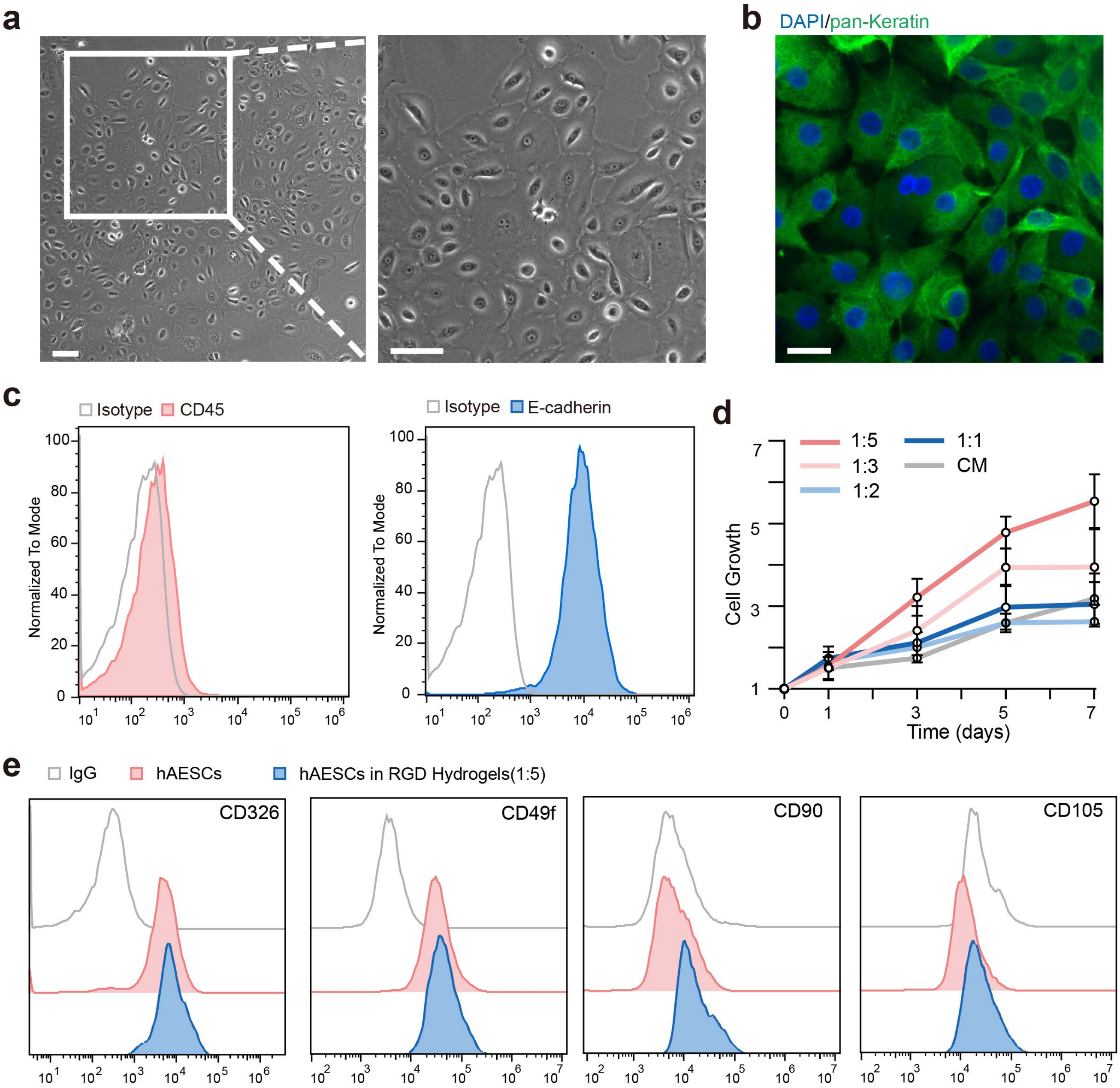

Figure. S1 Identification and Characterization of hAESCs a) Phase-contrast microscope image showing isolated hAESCs with cobblestone appearance. Scale bars = 100 μm. **b)** The representative epithelial marker pan-keratin detected by immunofluorescence microscopy. Scale bars = 50 μm. **c)** The hematopoietic markers CD45 and epithelial marker E-cadherin were determined using flow cytometry.

d) Viability of hAESCs within arginine–glycine–aspartic acid (RGD) hydrogels at different dilution for 1, 3, 5 and 7 days, assayed via cell counting kit-8 assay. Data are represented as mean ± SEM, n=3. **e)** Flow cytometry analysis of epithelial marker CD326 and CD49f, mesenchymal markers CD90 and CD105 in hAESCs with or without hydrogels culture for 72 h.

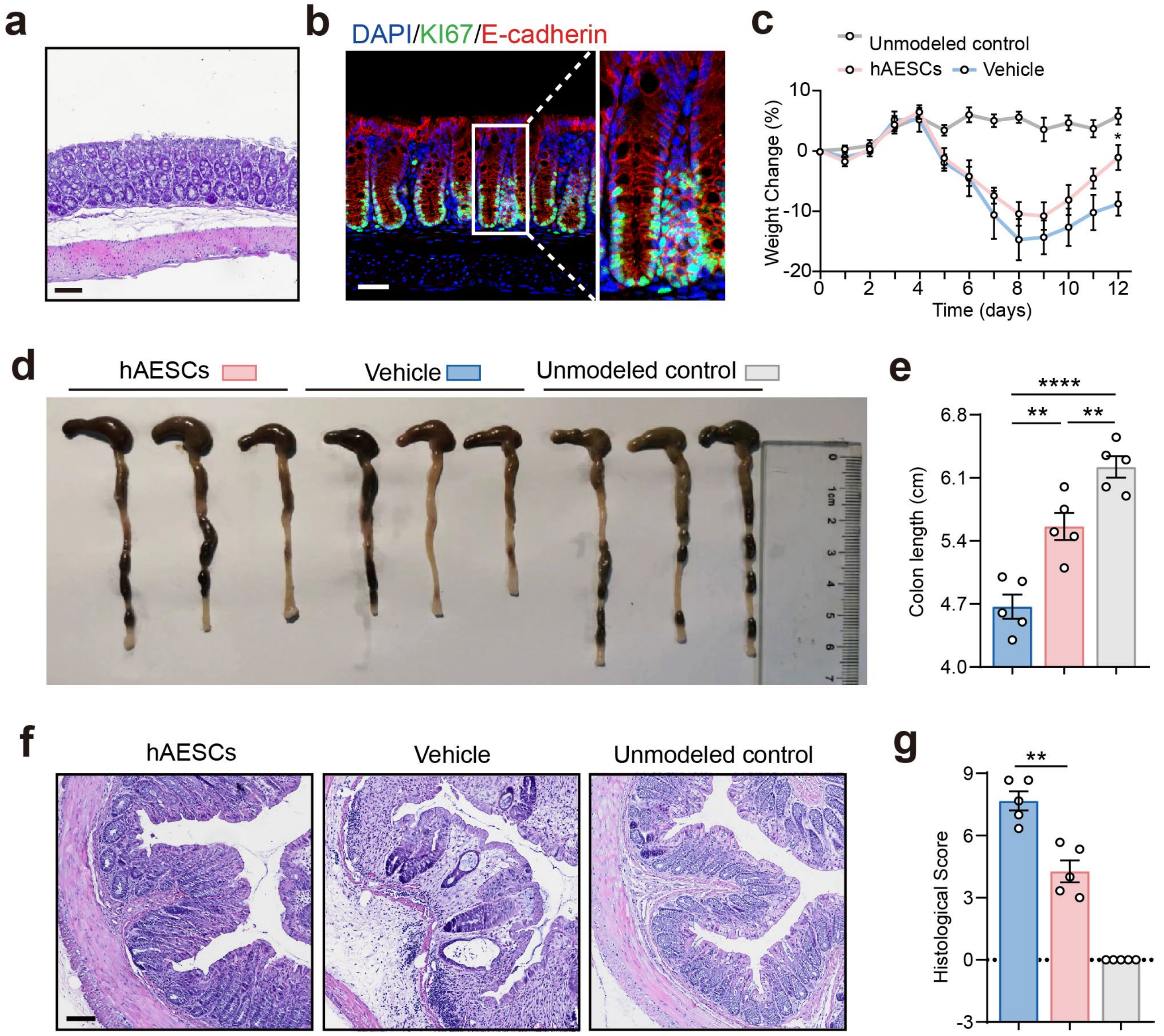

Figure. S2 Enhanced recovery of DSS-induced colitis in female mice following hAESCs treatment. a) Hematoxylin and eosin (H&E) staining of colon tissue sections of unmodeled mice. Scale bar=100μm. **b)** Ki67 (green) immunofluorescence staining of colon tissue sections of unmodeled mice. Scale bar=50μm. **c)** Changes in body weights during DSS-induced colitis and recovery in female

mice. n=6. **d, e)** Visual assessment of colon morphology (c) and quantification of the colon length at day 12 (e). n=5. **f, g)** Hematoxylin and eosin (H&E) staining of colon tissue sections (f) and assessment of histological scores (g). Scale bar=100μm. One-way ANOVA, followed by Tukey post hoc test or unpaired t test was performed. n=5. ****$p < 0.0001$; **$p < 0.01$; *$p < 0.05$.

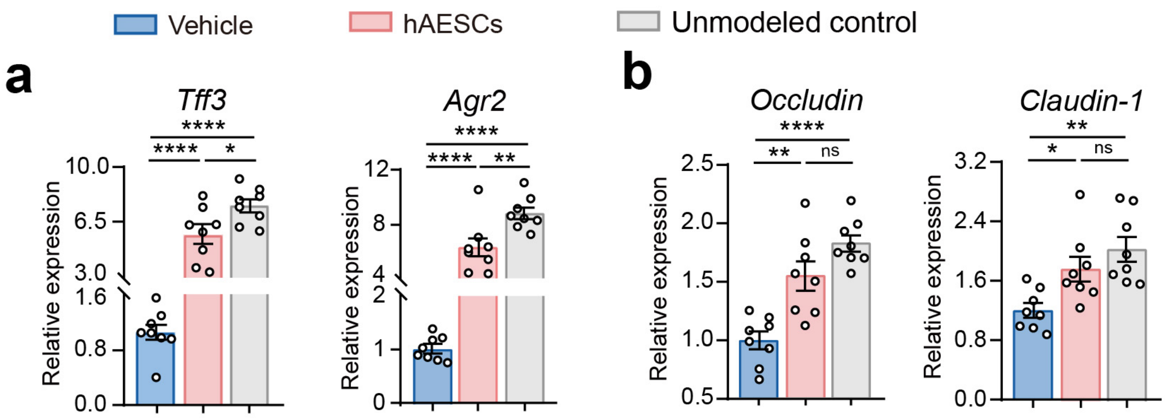

Figure S3. Mitigation of mucosal barrier injury in colon of mice by hAESCs treatment following DSS-induced colitis. Quantification of mRNA expression levels of indicated genes in the colonic tissues of mice subjected to DSS administration. Data are represented as mean ± SEM. One-way ANOVA, followed by Tukey post hoc test. n=8; ****$p < 0.0001$; ***$p < 0.001$; **$p < 0.01$; *$p < 0.05$; ns, not significant ($p > 0.05$).

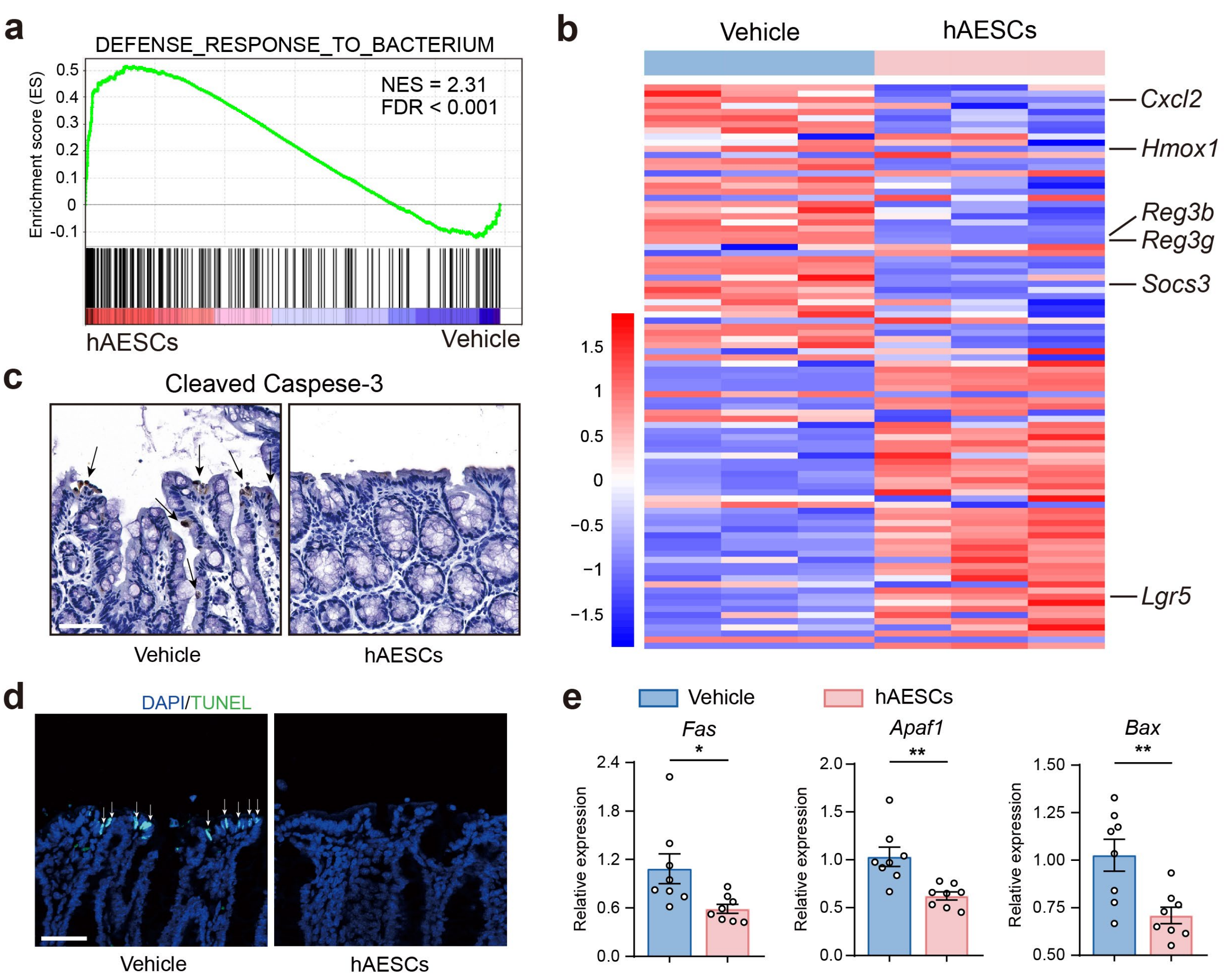

Figure S4. Mitigation of epithelial injury in colon of mice by hAESCs treatment following DSS-induced colitis. a) Gene set enrichment analysis (GSEA) revealing up regulated pathways in hAESCs-treated mice. **b)** Heat-map representation of mRNA expression levels in colonic epithelia from hAESCs-treated and untreated mice following DSS-induced colitis. The colors on the heat map reflect gene expression values normalized based on the mean expression of mRNA across all samples: blue indicates down-regulation, while red indicates up-regulation in the tissue. **c-e)** Examination of apoptotic epithelial cells in the colonic mucosa of mice with DSS-induced colitis, with or without subsequent hAESCs treatment, through cleaved-caspsec-3 immunohistochemistry staining (c), TUNEL assay (d), and qRT- PCR analysis of indicated genes (e). Scale bars = 50 μm. Data are represented as mean ± SEM, and unpaired t test was performed. n=8. $^{**}p < 0.01$; $^{*}p < 0.05$.

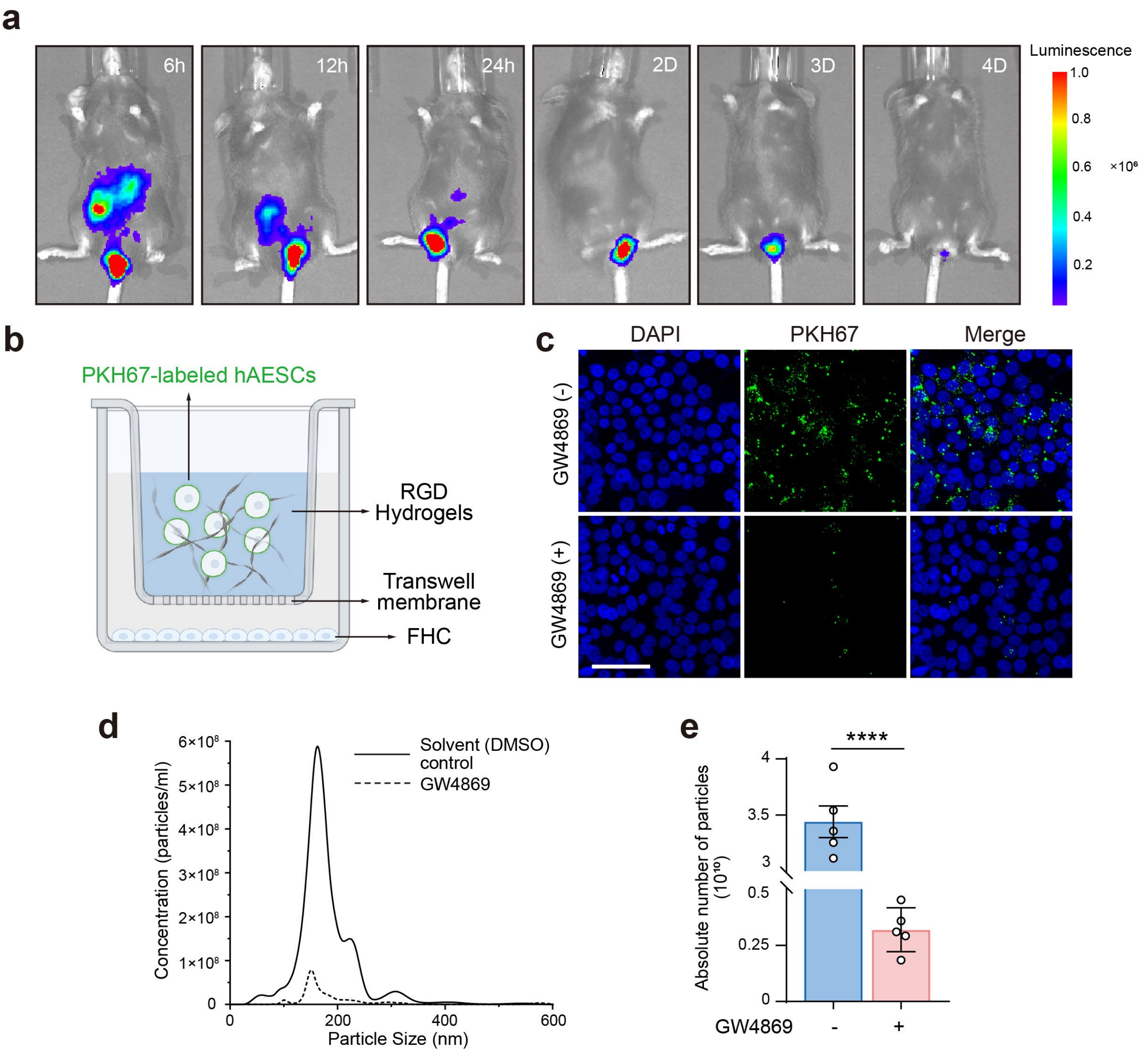

Figure S5. Release of hAESCs and exosomes from the RGD hydrogel. a) Representative bioluminescent images of hAESCs *in vivo*. The hAESCs, infected with a lentivirus carrying a firefly luciferase gene and encapsulated in RGD hydrogel, were injected into the colons of mice previously exposed to DSS in their drinking water. Bioluminescent imaging was conducted at various time points post-injection. **b)** Schematic diagram for the transwell assay. PKH67-labeled hAESCs were 3D cultured in the RGD hydrogel in the upper chamber of the transwell. These cells were co-cultured with FHC (fetal human colon) cells on the opposite side of the transwell membrane for 24 hours, with or without GW4869 (an exosomal inhibitor, 0.1 mM). **c)** FHC cells were imaged using confocal

microscopy, and the cell nuclei were stained with blue fluorescence (DAPI). Scale bars = 20 μm. **d)** Exosomes were extracted from the cell culture supernatant of 1×10^6 hAESCs cultured in RGD hydrogel for 48 hours. The isolated exosomes were resuspended and then diluted 1:100. The concentration of exosomes was measured using NTA. **e)** Cumulative data showing the absolute number of exosomes secreted from 1×10^6 hAESCs are presented. Data are represented as mean ± SEM, and unpaired t test was performed. n=5; ****$p < 0.0001$.

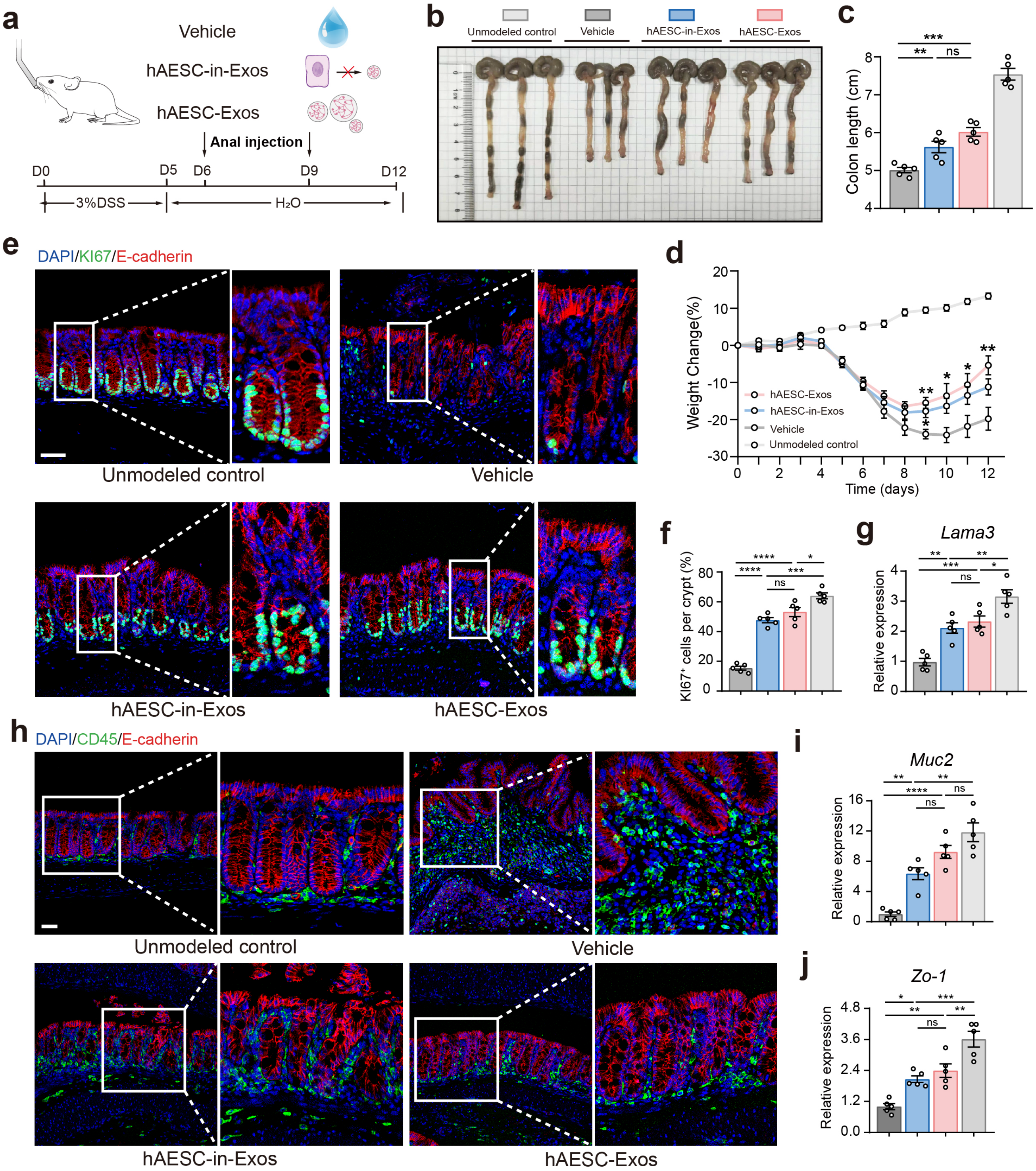

Figure S6. Enhanced recovery of DSS-induced colitis in mice following hAESC-Exos treatment. a) Schematic representation of injection in the mice with DSS-induced colitis. **b,c)** Assessment of colon appearance (b) and analysis of colon length from each group (c). **d)** Changes in body weights in mice during DSS-induced colitis and recovery. **e, f, g)** Evaluation of regenerated

epithelial cells in the colonic mucosa of mice with DSS-induced colitis using Ki67 immunofluorescence staining (e), with a quantification summary (f), and qRT-PCR analysis of indicated genes (g). Scale bars = 50 μm. **h)** Assessment of inflammatory cell infiltration in the colonic mucosa of mice with DSS-induced colitis employing CD45 (green) immunofluorescence staining. Scale bars = 50 μm. **i, j)** qRT-PCR analysis of indicated genes. Data are represented as mean ± SEM. One-way ANOVA, followed by Tukey post hoc test. n=5; ****$p < 0.0001$; ***$p < 0.001$; **$p < 0.01$; *$p < 0.05$; ns, not significant ($p > 0.05$).

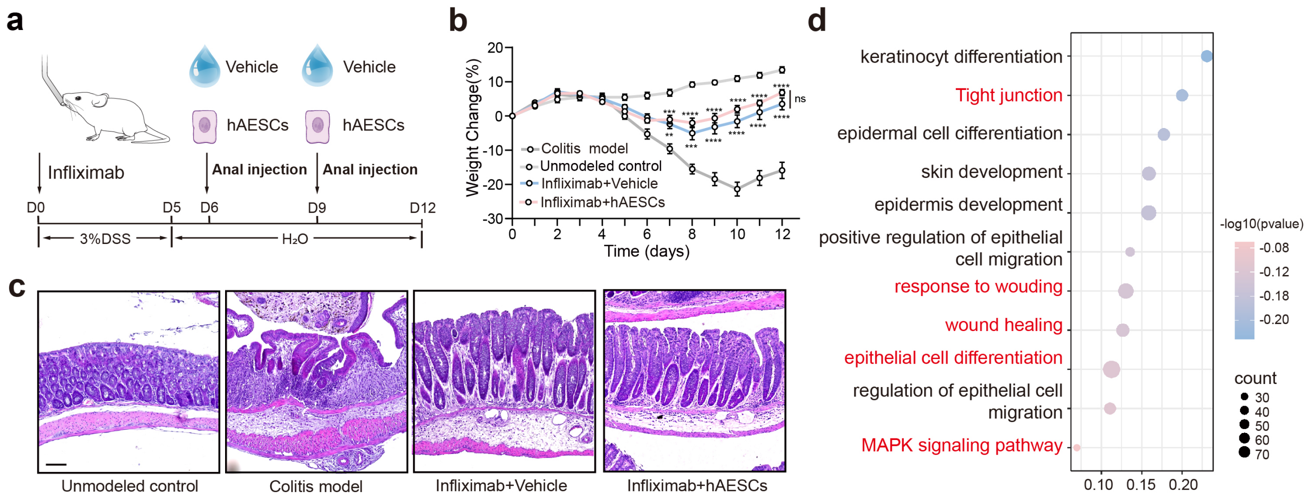

Figure S7. The potential effect of hAESCs in colitis mice following TNF signaling inhibition. **a)** Schematic representation illustrating the treatment protocol in the TNF signaling-inhibited murine colitis model. **b)** Changes in body weights during DSS-induced colitis and recovery in mice. Data are represented as mean ± SEM, n=8. **c)** Hematoxylin and eosin (H&E) staining of colon tissue sections. Scale bar = 100μm. **d)** Bubble map of KEGG pathway enrichment analysis of the highly expressed genes identified in the proteomic analysis of the hAESCs culture supernatant, associated with the repair of colonic epithelial injury. Data are represented as mean ± SEM. One-way ANOVA, followed by Tukey post hoc test. n=8; ****$p < 0.0001$; ***$p < 0.001$; **$p < 0.01$; ns, not significant ($p > 0.05$).
